# Comanagement of surgical patients between neurosurgeons and internal-medicine clinicians: observational cohort study

**DOI:** 10.1007/s11739-025-03866-x

**Published:** 2025-02-11

**Authors:** Ombretta Para, Joel Byju Valuparampil, Irene Merilli, Lorenzo Caruso, Asim Raza, Alberto Parenti, Carolina Angoli, Mohammed Al Refaie, Marzia Onesto, Lorenzo Barbacci, Carlo Nozzoli, Alessandro Della Puppa

**Affiliations:** 1 Internal Medicine 1, University Hospital of Careggi, Florence, Italy; 2 Clinical and Experimental Medicine and Medical Humanities, University of Insubria, Varese, Italy; 3 Neurosurgical Department, University Hospital of Careggi, Florence, Italy

**Keywords:** Neurosurgery, Comanagement, Hospitalist, Comorbidities

## Abstract

The rising prevalence of chronic diseases have contributed to a population with high complexity of care. There has been an increasing need for a new organizational model based on the interaction in the same department between the specialist skills of surgical and medical disciplines. This study aims to describe the implementation of a hospitalist co-management program in a Neurosurgery Department (ND) and its impact on the incidence of medical complications, 30 days readmission rate for medical causes, number of transfers to Intensive Care Units (ICU)/Neurosurgical Intensive Care Unit (NICU) or to medical wards (MW), length-of stay (LOS), mortality and satisfaction of health workers. We conducted an observational study comparing changes before and after the Internal medicine-Neurosurgical Comanagement (INC) intervention. We conducted a retrospective evaluation of patients enrolled before the INC intervention and a prospective evaluation of those enrolled after the INC intervention was implemented. We defined the pre-INC intervention group as 380 patients admitted to the ND for neurosurgical disease between January 2022 and April 2022 and the post-INC intervention group as 367 patients admitted to the ND between January 2023 and April 2023. INC intervention was associated with a significant decrease in medical complications during the hospital stay (OR 0.52; 95% CI; 0.39–0.70, *p* < 0.001), 30 days in-hospital readmission for medical reasons (OR 0.95; 95% CI 0.93–0.97, *p* < 0.001) and numbers of transfers to ICU/NICU (OR 0.31; 95% CI; 0.17–0.55, *p* < 0.001) or MW (OR 0.51; 95% CI 0.33–0.77, *p* = 0.002). During the INC intervention period, we observed a high satisfaction rate in health workers, evaluated by standardized questionnaire. In our study, LOS, in-hospital mortality and 30-day mortality were not significantly associated with INC. Hospitalist co-management in Neurosurgical Departments was associated with a reduced incidence of medical complications, 30-days in-hospital readmission and numbers of transfers to ICU/NICU or MW with a high satisfaction rate among healthcare workers, but without a significant decrease in LOS and mortality rate.

## Background

The increase in the average age of the population, the rising prevalence of chronic diseases and innovations in the pharmaceutical field have contributed to a population of hospitalized patients with high complexity of care [1–3]. Surgical patients are increasingly elderly, undergoing polypharmacy, suffering from multiple comorbidities that may exacerbate during the perioperative phase, and sometimes with social issues related to difficult home management [3]. Therefore, there has been an increasingly stringent need for a new organizational model based on the interaction in the same department between the specialist skills of surgical and medical disciplines. In this setting, the responsibility and the care burden of the patient are shared among different specialist figures, overcoming the consultative model that leads to the inevitable fragmentation of care. Indeed, the comanagement model that involves the integrated and shared management of the patient by medical and surgical specialists is becoming increasingly widespread. Between 2001 and 2006, there was an observed increase in the application of this organizational model by over 11% in the United States [4]. In the literature, the role of the hospitalist is often covered by the internal medicine specialist who, thanks to his cross-disciplinary skills and a holistic approach, seems to be the most suitable figure to ensure adequate perioperative care for complex patients [5–8]. Literature data [5, 9] show that internist-surgical comanagement seems to offer the opportunity to improve the care of patients undergoing surgery, allowing the different professional figures to focus more on their area of expertise. In particular, comanagement appears to be associated with a reduction in medical complications, the duration of hospital stays, the number of readmissions for medical complications, the number of specialist medical consultations required, and postoperative mortality rates [10–13]. Comanagement would also lead to a reduction in costs for the national health system and improved satisfaction of the healthcare personnel [13–17]. However, most data have been extrapolated from settings such as general surgery and orthopedics [4]. There is little data relating to the co-management carried out by the internist in the neurosurgery setting where the figure of the hospitalist is often covered by the neurologist [18].

The purpose of this study is to examine whether the internal medicine-neurosurgical comanagement (INC) intervention in the Neurosurgery Department is associated with a decrease in the number of medical complications. We also examine whether the INC intervention in Neurosurgery Department is associated with reduction in 30-days readmission rate for medical cause, number of transfers to Intensive Care Units (ICU)/Neurosurgical Intensive Care Unit (NICU) or to medical wards (MW), length-of-stay (LOS), in-hospital and 30-days mortality and satisfaction of hospital staff involved.

## Methods

A preliminary analysis of this population has been recently published as a research letter in the EJIM [6]. In the current study, we provide a more comprehensive and detailed analysis, with additional data and different endpoints, allowing for a more in-depth exploration of our findings.

### Study design and sample

We conducted a single-center before and after study comparing changes before and after the INC intervention. We defined INC intervention as the implementation of INC in the Neurosurgery department in January 2023.

### Setting and patients

This study was conducted at an academic medical center of neurosurgery mainly treating brain diseases. We included patients admitted to the Neurosurgical department for a neurosurgical disease, both for elective Neurosurgical surgery and emergencies. We excluded all patients who underwent stereotaxic intracranial radiosurgery, as this is performed in 1-day surgery. Our total study population comprised 747 patients. The Neurosurgical department of our academic medical center has 35 beds for patients hospitalized with diseases of neurosurgical interest.

We defined the pre-intervention (or pre-INC) group as 367 patients admitted to the Neurosurgery department between January 2022 and April 2022. We defined a post-intervention (or post-INC) group as 380 patients admitted to the Neurosurgery department between January 2023 and April 2023. During the pre- and post-study period, there were no other major organizational changes in the Neurosurgical department except for the implementation of comanagement.

### Outcome

Our primary composite outcome included medical complication**s,** number of transfers to ICU/NICU or to MW (internal medicine or geriatric or neurological wards), 30-days readmission rate for medical cause, LOS, in-hospital and 30 days mortality and satisfaction of hospital staff involved.

A “medical complication” was considered if that medical condition was not present on admission.

**Fig. 1:**
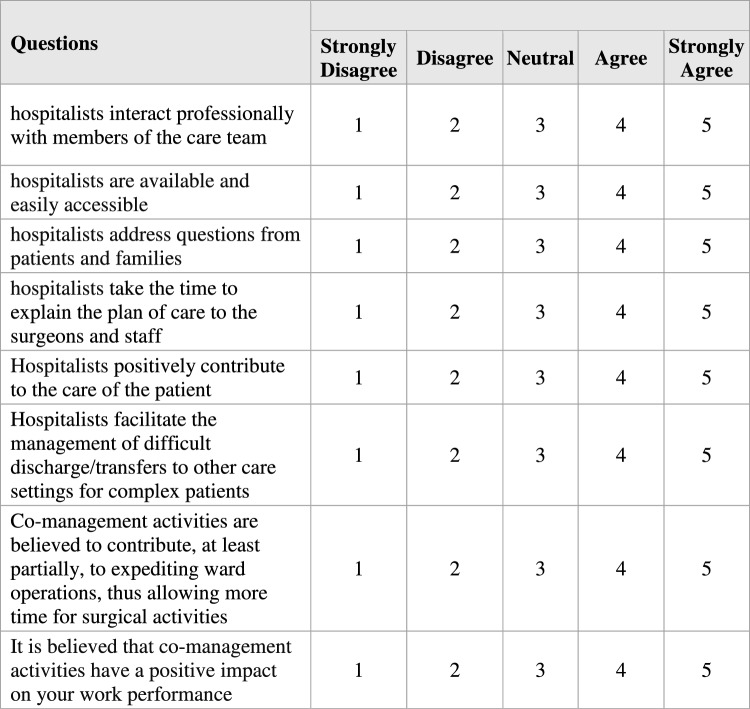
Questionnaire assessing healthcare personnel satisfaction regarding co-management activities [12]

As “medical complication” we considered: fever considered as body temperature > 37,8° [19]; respiratory failure considered as severe hypoxemia (PaO2 < 60 mmHg) with or without hypercapnia [20]; sepsis according to definition of 2021 Surviving Sepsis Campaign guidelines [21] anemia considered for hemoglobin < 11.5 g/dl (119 g/L) or hematocrit < 35% in female or hemoglobin < 13.5 g/dl (136 g/L) or hematocrit < 40% in males [22]; thrombocytopenia defined for platelet cut-off < 150,000/microL (< 150 × 109/L) [23] renal impairment defined according to KDIGO guidelines 2012 definition [24], heart failure defined according to 2021 ESC guidelines [25, 26] electrolyte disorders considered as hyponatremia (cut-off sodium concentration < 130 mEq/L) hypokalemia (cut-off potassium concentration < 3.0 mEq/L) and hypocalcemia (cut-off calcium concentration < 8,5 g/dl); Venous Thromboembolic Diseases [27] (clinical suspicion based on clinical signs, confirmed diagnostically via ultrasound), arrhythmias considered as ventricular and/or supraventricular arrhythmias; hypertension considered as elevated blood pressure episode occurring during the hospital stay according to ESH guidelines 2023 [28], infection of urinary trait; pneumonia; hyperglycemia defined as > 140 mg/dL (7.8 mmol/L) by the American Association of Clinical Endocrinologists and the American Diabetes Association [29]. We included only those medical complications that could be impacted by hospitalists during hospitalization. Medical complications and medical readmissions were assigned after internal medicine specialist revision of the hospital chart. This study was not powered to study surgical complications. We evaluated readmission to medical specialties to our hospital within 30-days from discharge; any planned readmission and any readmission for neurosurgical complications were excluded. Data were obtained on healthcare workers' satisfaction post-INC group using a questionnaire based on Likert scale [12] integrated with specific information about the utilization setting (Fig. 1). The questionnaire was submitted to 30 medical and nursing staff in the Neurosurgical Ward, divided into 9 tenured surgeons, 8 doctors in specialist training, 12 nurses and 1 physiotherapist.

### Data collection

For the patients' recruitments, we used a computerized electronic medical record (Archimed® medical software version 6.20 by B. Dannaoui, Florence, Italy).

We conducted a retrospective evaluation of patients enrolled before the intervention and a prospective evaluation of those enrolled after the intervention was implemented.

We collected data about the neurosurgical intervention (type and duration of surgery), hospitalization (LOS, number of internistic consultants, number of other type of consultants, execution of screening rectal swab for multidrug resistant (MDR) bacteria, need for transfusion of blood derivative products, medical complications) and outcome (home discharge or transfer to structures of social and health care, transfer to MW or to ICU/NICU, death). Comorbidities profile was evaluated by Charlson Comorbidity Index. We also collected data about home therapy, particularly about therapeutic complexity. We reported the presence of home therapy with insulin, anticoagulant and/or antiaggregant agents which are an additional risk factor for possible postoperative complications [30–33].

Our study also included a 30-days follow-up from the date of discharge: we noted eventual re-hospitalization or death, reason of the re-hospitalization or reason of death (generically divided into medical or surgical reasons). Data for 30-days mortality and all hospital-readmissions were acquired by hospital data or phone calls for all patients. Any hospitalization of the patient within 30 days post-surgery, after being discharged home, was considered readmission.

The study was performed in accordance with the Declaration of Helsinki and local regulations. The protocol was approved by the Ethics Committee of our center, University Hospital of Careggi, Florence. The authors declare they have no conflict of interest.

### Structure of ICN and neurosurgery ward organization

We have a model in which the same two hospitalists were dedicated to Neurosurgery from Monday to Saturday in the morning. The hospitalist is a specialist in Internal Medicine trained in managing perioperative patients. In our organizational model there were two hospitalists dedicated to the comanagement project and they were residents from the Internal Medicine department situated near Neurosurgery. Due to organizational needs, they performed afternoon and night shifts in the Internal Medicine ward. Therefore, during night or holiday shifts, patients admitted in Neurosurgery ward are managed by neurosurgeons who can request consultation from the anesthetist located on the lower floor in case of emergency or from the internist located in the Internal Medicine ward near Neurosurgery.

In consideration of the shifts, the two hospitalists were not always both present in the morning. The number of patients taken care of by the hospitalist changes every day, based on the type of patients admitted and the possible development of unexpected complications. The hospitalist is autonomous in diagnostic and therapeutic decisions but shares the decision-making process with the neurosurgeons.

Every morning from Monday to Saturday each patient admitted to Neurosurgery ward was discussed on multidisciplinary rounds with neurosurgeons, nurses, physiotherapists and speech therapists. The hospitalist actively participates in these multidisciplinary meetings where internal medicine problems and risk factors are highlighted. Furthermore, hospitalists performed bedside echography and ultrasound-guided positioning of intravenous catheterization.

The neurosurgery ward consists of 27 beds and the average LOS is about 9 days. About beds turnover in our Neurosurgery, on average 3 discharges and 3 admissions are performed each day. The ward usually admits patients for both elective and emergency neurosurgical intervention. Typically, about 40% are patients admitted as emergency cases and 60% as elective cases. Emergency admissions obviously cannot be planned and this often causes changes in the predefined surgical program.

### Statistical analysis

The study was carried out and reported according to the STROBE guidelines for observational studies [34]. In the traditional management group, the incidence rate for the primary endpoint (a composite of 30-day mortality or transfer to the ICU) was observed. To achieve clinical relevance, we aim for a one-third (33%) reduction in the primary endpoint. Based on this target, a minimum of 250 patients per group (traditional management and co-management) is needed to achieve a 5% significance level (two-sided). Considering a potential 2% rate of unusable data in each group, the sample size requirement increases to 255 patients per group, resulting in a total of 510 patients needed for the study.

The normality of data distribution was assessed using the Shapiro–Wilk test. Continuous variables were expressed as mean plus or minus standard deviation (SD) or as median with interquartile range (IQR), as appropriate. Categorical data were reported as counts and percentages. Categorical variables were compared using Chi-squared or Fisher’s test, as appropriate. Continuous variables were compared with Student’s test or Mann–Whitney U-test, when appropriate. Every variable associated with an outcome of the study with a *p*-value < 0.10 (entry level) was included in a multivariate binary logistic regression. Stepwise elimination was performed to finalize the independent predictors of the multivariate models. Statistical significance was reached when the p-value was < 0.05 (two-tailed). Results of the multivariate analyses were expressed as Odds Ratios (ORs) and the corresponding 95% Confidence Interval (CI). Statistical analyses were performed using STATA-16/MP (StataCorp LP, College Station, TX, USA).

## Results

Characteristics of patients in the pre- and post-ICN groups are reported in Table 1. There were no significant differences in the patient characteristics between the two groups in terms of sex, age, autonomy profile identified with the Barthel Index Code and home therapy (Table 1).

**Table 1:** Baseline characteristics of patients in intervention and control groups

Baseline characteristics	Patient cohort with traditional management (380)	Patient cohort with integrated management (367)	p < 0.05
Sex male	213 (56.1%)	198 (54.0%)	0.607
Sex female	167 (43.9%)	169 (46.0%)	0.607
Mean age (years)	62.88 ± 17.95	63.29 ± 16.90	0.751
Age > 75 years	121 (31.8%)	115 (31.3%)	0.937
Mean barthel index Code	55.32 ± 39.32	57 ± 37.76	0.550
Barthel index code < 60	196 (51.6%)	186 (50.7%)	0.826
Insulin therapy	13 (3.4%)	13 (3.5%)	1.000
Anticoagulant therapy	40 (10.5%)	47 (12.8%)	0.362
Antiplatelet therapy	52 (13.7%)	27 (15.5%)	0.534
History of arterial hypertension	140 (36.8%)	145 (45.0%)	0.026
History of localized solid tumoral lesion	45 (11.8%)	104 (28.3%)	0.000
History of cognitive Decline	20 (5.3%)	26 (7.1%)	0.361
History of coronary artery disease	9 (2.4%)	31 (8.4%)	0.000
History of diabetes mellitus	35 (9.2%)	39 (10.6%)	0.542
Mean charlson comorbidity index	2.47 ± 2.40	3.48 ± 2.54	0.000
Charlson comorbidity index > 5	41 (10.8%)	70 (19.1%)	0.002

About comorbidities (Table 1), Charlson Comorbidity Index was significantly higher (*p* < 0.001) in the cohort of patients post-INC.

Table 2 shows the data on the admission diagnoses of the two cohorts under study. Table 3 shows the data about intervention of the two cohorts under study. Brain tumor resection and evacuation surgery were performed significantly more frequently in the patients group pre-INC (*p* = 0.036 and *p* = 0.032, respectively) and endovascular repair or occlusion of head and neck vessels was performed significantly more frequently in the patients group pre-INC (*p* = 0.007).

**Table 2:** Admission diagnoses in the two study populations

Admission diagnoses	Patient cohort with traditional management (380)	Patient cohort with integrated management (367)	*p* < 0.05
Intracranial tumor	133 (35.0%)	161 (43.9%)	0.014
Post-Traumatic Chronic Subdural Hematoma	59 (15.5%)	54 (14.7%)	0.761
Intraparenchymal hemorrhage	42 (11.1%)	14 (3.8%)	0.000
Hydrocephalus	30 (7.9%)	40 (10.9%)	0.169
Bleeding Aneurysm	25 (6.6%)	20 (5.4%)	0.542
Acute subdural hematoma	21 (5.5%)	17 (4.6%)	0.349
Unruptured Aneurysm	12 (3.2%)	7 (3.2%)	0.354
Others*	58 (15.3%)	54 (14.7%)	0.838

*Others: Cerebrospinal Fluid Collection, Spasticity, Surgical Wound Complications, Brain Abscess, Hemorrhagic Infarct, Arnold-Chiari Malformation, Post-Traumatic Skull Operculum Depression, Extradural Empyema, Craniolacunia Post-Decompressive Craniotomy, Baclofen Pump Replacement, Spinal Neurinoma, Vertebral Fracture, Headache of Undetermined Nature, Arteriovenous Malformation (AVM), Implant Complications for Revision, IPG (Implantable Pulse Generator) Replacement, Cerebral Infarction, Cerebrospinal Fluid Hypotension Syndrome, Arachnoid Cyst of the Third Ventricle, Cerebrospinal Fluid Fistula, Spinal Cord Injury, Parkinson's Disease, Intracranial Hypertension Due to Infarction, Thalamic Hamartoma, Pharmacoresistant Epilepsy

**Table 3:** Types of surgical interventions performed in the two study populations

Treatment	Patient cohort with traditional management (368)	Patient cohort with integrated management (381)	*p* < 0.05
Brain tumour resection	113 (29.7%)	136 (37.1%)	0.036
Haematoma evacuation	93 (24.5%)	66 (18.0%)	0.032
VP Shunt/EVD (Ventriculoperitoneal Shunt/External Ventricular Drainage)	32 (8.4%)	39 (10.6%)	0.320
No surgical intervention	29 (7.6%)	33 (9.0%)	0.511
Posterior fossa surgery	34 (8.9%)	27 (7.4%)	0.255
Endovascular repair or occlusion of head and neck vessels	17 (4.5%)	4 (1.1%)	0.007
Diagnostic cerebral angiography	21 (5.5%)	13 (3.5%)	0.221
Others*	41 (10.8%)	49 (13.4%)	0.312

*Others: Implantation or Replacement of Intracranial Neurostimulator Electrodes, Meningeal Repair, VP Shunt Closure, Abdominal Pump Replacement, IPG (Implantable Pulse Generator) Replacement, Dorsal and Thoracolumbar Arthrodesis, Infusion Pump Implantation, Spinal Blood Patch, Surgery on the Spinal Cord and Vertebral Canal Structures, Intervertebral Disc Removal, Revascularization, Biopsy, Craniectomy, Aneurysm Clipping, Surgical Wound Dehiscence Repair, Surgical Site/Wound Revision, Cranioplasty

Table 4 shows results about composite outcomes. ICN intervention was associated with a significant decrease in medical complications during the hospital stay (OR 0.52; 95% CI; 0.34–0.70. *p* < 0.001). number of patients transferred to ICU/NICU (OR 0.31; 95% CI; 0.17–0.55. *p* < 0.001) or to MW (OR 0.51; 95% CI 0.33–0.77, *p* = 0.002), 30-days in-hospital readmission for medical reasons (OR 0.95; 95% CI 0.93–0.97, *p* = 0.000) and total 30-days in-hospital readmission (OR 0.30; 95% CI 0.15—0.57; *p* < 0.001). In our study, 30-days in-hospital readmission for surgical reasons was not significantly associated with INC (OR 0.61; 95% CI 0.29–1.26; *p* = 0.208). The average LOS was higher, though not significantly, in the co-management care model. Specifically, it increased from a mean value of 9.10 ± 7.51 days in the traditionally managed patient cohort to 10.03 ± 6.91 days in the integrated management patient cohort, but without statistical significance (*p* = 0.079).

**Table 4:** Outcomes of the study

Outcomes	Patient cohort with traditional management (380)	Patient cohort with integrated management (367)	OR	95% CI	*p* < 0.05
Medical complications	179 (46.8%)	116 (31.6%)	0.52	0.39–0.70	0.000
Number of transfers ICU/NICU	49 (12.9%)	16 (4.4%)	0.31	0.17–0.55	0.000
Number of transfers to MW	67 (17.6%)	36 (9.8%)	0.51	0.33–0.77	0.002
In-hospital readmission for medical reasons at 30 days	19 (5.0%)	0 (0.0%)	0.95	0.93–0.97	0.000
In-hospital readmission for surgical reasons at 30 days	20 (5.3%)	12 (3.3%)	0.61	0.29–1.26	0.208
In-hospital readmission for any reason at 30 days	39 (10.3%)	12 (3.3%)	0.30	0.15–0.57	0.000
Mean LOS	9.10 ± 7.51	10.03 ± 6.91			0.079
In-hospital mortality	6 (1.6%)	11 (3.0%)	1.93	0.71–5.26	0.226
30-days mortality	28 (7.4%)	23 (6.3%)	0.84	0.48–1.49	0.565
Adverse composite outcome	55 (14.5%)	27 (7.4%)	0.47	0.26–0.76	0.002

In-hospital mortality (OR 1.93; 95% CI 0.71–5.26; *p* = 0.226) and 30-day mortality (OR 0.84; 95% CI 0.48–1.49; *p* = 0.565) were not significantly associated with INC.

We also conducted a sub-analysis evaluating the adverse composite endpoint (considered as the need for transfer to ICU/NICU and/or in-hospital mortality) during hospitalization in INC intervention group: in our analysis, the INC intervention was significantly associated with a reduction in adverse composite outcome (OR 0.47; 95% CI 0.26–0.76; *p* = 0.002).

Table 5 reported medical complications observed during hospitalization. We can highlight a significant reduction in three complications: hyperglycemia (*p* = 0.012; OR 0.40; 95% CI, 0.20–0.83), electrolyte imbalance (*p* = 0.021; OR 0.44; 95% CI, 0.22–0.87) and arterial hypertension (*p* = 0.000; OR 0.15; 95% CI, 0.08–0.25).

**Table 5:** Medical complications observed in the two study populations

Medical complications	Patient cohort with traditional management (368)	Patient cohort with integrated management (381)	*p* < 0.05
Fever	44 (11.6%)	68 (18.5%)	0.010
Respiratory failure	18 (4.7%)	23 (6.3%)	0.423
Sepsis	0 (0.0%)	5 (1.4%)	0.028
electrolyte imbalance	27 (7.1%)	12 (3.3%)	0.021
Thrombocytopenia	3 (0.8%)	7 (1.9%)	0.216
Renal impairment	2 (0.5%)	11 (3.0%)	0.011
Cardiac impairment	17 (4.5%)	17 (4.6%)	1.000
Venous Thromboembolic Diseases	11 (2.9%)	12 (3.3%)	0.834
Arrhythmias	3 (0.8%)	5 (1.4%)	0.500
Hypertension	91 (23.9%)	16 (4.4%)	0.000
Infection of urinary trait	16 (4.2%)	8 (2.2%)	0.146
Pneumonia	23 (6.1%)	26 (7.1%)	0.658
Hyperglycemia	27 (7.1%)	11 (3.0%)	0.012

Detailed data on the satisfaction rate of the comanagement service are shown in Table 6*.* The evaluation of the “Strongly agree” responses to various parameters related to satisfaction with the comanagement service alone yielded a response rate of 63.8%.

**Table 6:** Results related to the questionnaire evaluating the satisfaction of neurosurgical healthcare personnel regarding co-management activities

Questions	Strongly Disagree	Disagree	Neutral	Agree	Strongly Agree
Hospitalists interact professionally with members of the care team	0 (0.0%)	0 (0.0%)	1 (3.3%)	6 (20.0%)	23 (76.7%)
Hospitalists are available and easily accessible	0 (0.0%)	1 (3.3%)	0 (0.0%)	10 (33.3%)	19 (63.3%)
Hospitalists address questions from patients and families	0 (0.0%)	1 (3.3%)	6 (20.0%)	8 (26.7%)	15 (50.0%)
Hospitalists take the time to explain the plan of care to the surgeons and staff	0 (0.0%)	0 (0.0%)	2 (6.7%)	10 (33.3%)	18 (60.0%)
Hospitalists positively contribute to the care of the patient	0 (0.0%)	0 (0.0%)	1 (3.3%)	7 (23.3%)	22 (73.3%)
Hospitalists facilitate the management of difficult discharge/transfers to other care settings for complex patients	0 (0.0%)	1 (3.3%)	4 (13.3%)	11 (36.7%)	14 (46.6%)
Co-management activities are believed to contribute, at least partially, to expediting ward operations, thus allowing more time for surgical activities	0 (0.0%)	1 (3.3%)	1 (3.3%)	10 (33.3%)	18 (60.0%)
It is believed that co-management activities have a positive impact on your work performance	0 (0.0%)	1 (3.3%)	1 (3.3%)	4 (13.3%)	24 (80.0%)

## Discussion

Our study shows that intervention by INC hospitalist was associated with a significant decrease in medical complications during hospitalization, transfers in ICU/NICU or MW, 30-days readmission for medical cause. Furthermore, the intervention by INC hospitalist was associated with a greatest satisfaction of healthcare workers. There was no significant difference in LOS, in-hospital and 30-days mortality.

Patients in post-INC cohort had a lower incidence of medical complications compared to the traditional cohort with a lower incidence of hypertension, hyperglycemia and electrolyte disorders. Huddleston J et al. [13] conducted a study on orthopedic patients with high perioperative risk, observing a significant reduction of medical complications after surgery with a significant decrease of minor complications like electrolyte disorders, fever and urinary tract infection. Similarly, Rohatgi N. et al. [12], in a neurosurgical and orthopedic study has proven the reduction of at least one medical complication during the hospitalization with comanagement model. This can be explained by an early identification of high-risk patients, for comorbidities and polytherapy, and by early management for possible internist issues.

In the literature, there are few studies about transfer in ICU or internistic setting with comanagement model. Della Rocca G. et al. [10] in a study conducted on geriatric patients with hip fracture have shown a significant reduction of patients that needed a transfer in ICU in the comanagement cohort. This result could be related to early and global management of difficult patients, with the opportunity of optimizing the diagnostic-therapeutic process during the stay in surgical ward.

About in-hospital readmission, there have been several smaller studies on INC [10, 13, 35–45]. Rohatgi N. et al. [12] shows a significant reduction in 30-days hospital readmission. This observational retro-prospective study on 20.625 patients spread on 5 years is one of the biggest studies in literature about comanagement. Shaw M. et al. [46] in a metanalysis has considered seven studies [10], [12], [35], [40], [41], [43], [44], about the impact of comanagement in five different surgical settings (neurosurgery, cardiac surgery, vascular surgery, orthopedic, general surgery), proving that there is no relation between the reduction of readmission in 30 days and the comanagement model. However, this study had a large heterogeneity (I^2^ heterogeneity index = 78%), with a wide CI (CI, 0.68–1.16). The relation between 30-day readmission for medical cause and the role of the hospitalist, introduced by Rohatgi N. et al. [12] and enlightened by our study, could be justified by a better diagnostic and therapeutic accuracy for patients with comorbidities in the surgical ward. Our population had a wide spectrum of comorbidities: 21,8% of patients had a Charlson Comorbidity Index greater than 5. The Charlson Comorbidity Index is higher in the post-intervention group: it could be a bias related to the retrospective data collection in pre-intervention group. Furthermore, neurosurgeons who compiled medical records and wrote patient histories often focus on surgical issues and all the patient's comorbidities were often not exactly reported in the records managed by neurosurgeons.

Also, 11.9% of patients were in therapy with anticoagulants or insulin, treatments that increase the complexity of the medical assistance during the recovery and this can expose the patient to an additional risk of developing internal medium-term complications.

Results about satisfaction of the neurosurgical staff shows that ICN provides an opportunity for healthcare workers in order to optimize daily work and to improve patient management. Rohatgi N. et al. [12] reported a value of 59.8% for the same parameter in orthopedic and neurosurgery. INC hospitalist coordinate decisions between consultants and other members of the care team. ICN also provides an opportunity for addressing several medical questions from the nurses, patients and families. In several surgery departments, hospitalists were an integral part of the Early Recovery After Surgery (ERAS) pathway for multimodal pain management, early mobilization, fluid management and optimization of nutrition post-operatively [47]. It could be useful to introduce this model also in the neurosurgical department.

In our study, the in-hospital mortality and the 30 days mortality are not significantly related to the comanagement model. The metanalysis of Shaw M. et al. [46], with the aim of evaluating the impact of comanagement in surgical setting, has considered 12 studies [10, 12, 13, 35, 38–45] without any significant relation between the reduction in-hospital mortality and the comanagement model. Specifically, six studies [10, 12, 38, 40, 41, 44] reported a difference in terms of mortality reduction between the post-INC group and the traditional management group, of which only one [40] showed a statistically significant difference (*p* = 0.049). Only the study by Huddleston et al. [13] did not find a difference in terms of mortality reduction between the two groups. Five studies [35, 39, 42, 43, 45] reported higher mortality in the post-INC group, with only one [42] showing a statistically significant difference (*p* = 0.036; OR 4.1; 95% CI, 1.1–15.7).

The Tadros R. et al. [11] study, conducted on 1059 patients treated with vascular surgery, has shown a significant reduction on in-hospital mortality in the cohort of the comanagement model. This result can be explained by the clinical features and comorbidities of the population. In fact, only patients with ASA score greater than 2 (ASA Physical Status Classification System) have been selected for the study. An ASA score greater than 2 matches to severe systemic disease and important functional limitation with one or more moderate–severe disease. In literature and in our study, the low impact of the comanagement model on in-hospital and 30 days-mortality could be explained by a greater impact of the surgical disease on this endpoint, compared to medical complications, usually minor complications, during the stay [9, 13].

Our study has several limitations. First, this is a single institution study at a University Hospital. Second, this is an observational study in which we can only report associations and there could be unobserved variables which bias between group comparison. Furthermore, reviewers did not blind the complications in the two periods. Third, we analyzed a restricted population and restricted period. Fourth, the two populations had significant differences in comorbidities and this can be a bias for endpoints such as LOS. LOS could certainly be a critical outcome to understand the broader implications of this care model, not only in clinical terms but also in terms of healthcare costs. There was not a significant difference in LOS between two periods. A possible explanation could be related to differences in the comorbidity profile between the study group and the control group. Indeed, it can be observed that the population in the integrated management cohort during the study period had a significantly more complex comorbidity profile (Table 1). This may have been associated with an increased care burden and a greater need for subsequent transfer to long-term care or rehabilitation facilities, leading to an inevitable increase in the length of stay, not so much due to clinical conditions, but rather to logistical factors. The average waiting time for transfer to a facility was approximately seven days. Additionally, the finding of an increased length of stay may be consistent with the results of our study showing a reduction in ICU transfers, medical ward transfers, and 30-day readmissions for medical reasons. The role of a hospitalist in the neurosurgery department may have led to an increased length of stay in favor of a more stable clinical condition at discharge, with reduced use of medical ward beds.

Another limitation concerns the selection of the results used to evaluate patients in the pre- and post-intervention groups: specifically, the inclusion of arterial hypertension among the complications assessed in the primary composite outcome could represent a significant limitation. The study design—retrospective for the pre-intervention group and prospective for the post-intervention group—along with the broad range of clinical nuances associated with the term "hypertension", which spans from blood pressure values slightly above normal limits to hypertensive emergencies, may have influenced the analysis of this outcome.

Finally, one of the limitations of the study is the retrospective nature of the study and that prospective studies would be desirable to validate the results.

Our study has several strengths. To our knowledge, this is one of the first studies reporting the impact of INC hospitalist by internist on the outcomes of Neurosurgery patients. The study reports outcomes that are relevant to providing better quality care. This study also shows the importance of multidisciplinary management and coordination between care teams to improve outcomes of neurosurgery patients.

## Conclusion

In conclusion, this is one of the first studies on INC in Neurosurgery to our knowledge reporting the association of the internist intervention in neurosurgical department with important clinical outcomes such as perioperative medical complications, number of transfers to ICU/NICU or MW, 30-day readmission for medical cause and healthcare workers satisfaction**.** However, by mean of these preliminary data and due to the monocentric and retrospective nature of the sample, it was not possible to demonstrate a reduction in LOS and mortality following the comanagement intervention. Growing number of complex patients with a lot of comorbidities makes a new management model necessary for improving quality and well-coordinated care. It is necessary to interpret our results with greater caution, considering the retrospective nature and limitations of our study. Further studies are required to prove clinical and economic benefits of this comanagement system.
